# Can *Trichoderma harzianum* be used to enhance the yield and nutrient uptake of *Lactuca sativa* cv “Lollo Rosso” in floating systems?

**DOI:** 10.1002/fsn3.4127

**Published:** 2024-03-25

**Authors:** Edris Shabani, Naser Alemzadeh Ansari, Mohammad Reza Fayezizadeh, Matteo Caser

**Affiliations:** ^1^ Department of Horticultural Science, Faculty of Agriculture Shahid Chamran University of Ahvaz Ahvaz Iran; ^2^ Departments of Agricultural, Forest and Food Sciences University of Torino Grugliasco Italy

**Keywords:** biostimulants, fungi, hydroponic, lettuce, nutrition, phosphorus

## Abstract

An experiment was performed to evaluate the effect of *Trichoderma harzianum* MVT801 combined with different ratios of nutrient solution (NR) (25%, 50%, and 100%) on the growth and physiological traits of *Lactuca sativa* “Lollo Rosso” plants cultivated in floating systems. Inoculation of lettuce plants with *T. harzianum* MVT801 (T_1_) in a floating system improves biometric properties, photosynthetic parameters, and nutrient uptake compared with uninoculated treatment (T_0_). The results clearly showed that in T_1_, despite a 50% reduction in the ratio of nutrient solution, no significant difference was observed in the growth and photosynthesis characteristics and nutrient uptake in *L. sativa* “Lollo Rosso” leaves compared with a complete nutrient solution treatment (100%), which is one of the notable results of this study. In this regard, the highest yield was observed in T_1_NR_50_ (inoculated with fungi and 50% of the nutrient solution) and T_1_NR_100_ (inoculated with fungi and complete nutrient solution) treatments. Also, the highest concentrations of phosphorus and potassium in “Lollo Rosso” leaves were observed in T_1_NR_50_ and T_1_NR_100_ treatments. Accordingly, the use of *T. harzianum* in floating lettuce cultivation could be recommended to increase crop productivity and reduce the use of chemical fertilizers.

## Graphical abstract

This experiment investigated the effect of *Trichoderma harzianum* (T1) combined with different ratios of nutrient solution (NR) (25 (NR25), 50 (NR50), and 100% (NR100)) on the growth and physiological traits of “Lollo Rosso” lettuce plants cultivated in a floating system. The highest yield and the highest concentrations of elements were observed in the T1NR50 and T1NR100 treatments. Accordingly, the use of *T. harzianum* in floating lettuce cultivation could be recommended to increase crop productivity and reduce the use of chemical fertilizers.

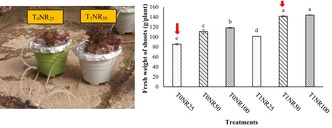

## 1 INTRODUCTION

The consumption of *Lactuca sativa* (lettuce) “Lollo Rosso” has recently attracted the attention of producers and consumers due to its attractive appearance and high amounts of bioactive molecules such as anthocyanins, minerals, and phenolic compounds (Sublett et al., 2018). Lettuce is ranked fifth in the world after corn, rice, potato, and tomato in terms of cultivation area (Aghabeygi et al., 2017). Previous findings showed that world lettuce production has grown by 118% in the last two decades (Aghabeygi et al., 2017). According to the US Agricultural Census information in 2012, lettuce ranked first in the total market value of fresh vegetables. In temperate countries, lettuce cultivation in polyethylene plastic tunnels is expanding due to increased seasonal availability and increased crop quality (García‐Macías et al., 2007). In Iran, the total greenhouse and farm production of lettuce on 17,000 hectares is about 540,000 tons (Ghotbi et al., 2020).

Growing vegetables in greenhouses and plastic tunnels can be a more efficient option for land use and other resources such as water and energy (Rahi, 2016). Another advantage of greenhouse cultivation is a 31% reduction in water consumption compared to the farm (Rahi, 2016). On the other hand, in recent years, rising production costs, such as the supply of chemical fertilizers, have posed severe challenges to greenhouse production. Therefore, it seems necessary to provide practical solutions to reduce the consumption of agricultural inputs, reduce environmental risks, and increase production efficiency.

Currently, the growing global population is expected to reach 10 billion by 2050. It is necessary not only to increase agricultural productivity but also to solve the problem of widespread use of chemical products (i.e., fertilizers and pesticides) and their negative impact on the environment and human health (Rouphael & Colla, 2020). A promising and environmentally friendly strategy for agriculture is the integrated use of various non‐chemical products in cropping systems, including the use of plant biostimulants (PBs) based on beneficial microorganisms and molecules of natural origin (Caser et al., 2019, 2020; Witkowicz et al., 2019). PBs are constituted of various bioactive substances, formulations of compounds or microorganisms, such as humic and fulvic acids, microalgae extracts, protein hydrolysis, chitosan, silicon, as well as mycorrhizal fungi, and plant growth‐promoting rhizobacteria (PGPR) that can improve plant growth, strength, and yield even in non‐optimal cultivation conditions (Carillo et al., 2020; Colla et al., 2015; Fiorentino et al., 2018; Rouphael et al., 2020; Stelluti et al., 2023). Also, other beneficial microorganisms, such as *Trichoderma* spp., resulted in improved plant growth, yield, and quality by controlling pests and pathogens, increasing nutrient efficiency, and simultaneously regulating maximal photosynthetic capacity and carbohydrate metabolism (Morán‐Diez et al., 2020; Vinale et al., 2008).

Previous research has shown that various strains of *Trichoderma* spp., such as *Trichoderma harzianum*, *Trichoderma asperellum*, *Trichoderma viride*, *Trichoderma virens*, and *Trichoderma atroviride*, act as PBs, mainly by stimulating the efficiency of nutrient utilization, which in turn will increase plant vigor and abiotic stress tolerance (Colla et al., 2015; Fiorentino et al., 2018; López‐Bucio et al., 2015; Rouphael et al., 2020). Rouphael et al. (2020) showed that, in greenhouse and open field cultivation of lettuce, the use of *T. virens* increased yield, potassium concentration, total dry weight, CO_2_ uptake rate, and reduced nitrate content and leaf stomata resistance. Lettuce grown in vermicomposts mixed with *T. asperellum* in pot cultivation also showed the highest plant height, number of leaves, leaf area, stem cross section, and dry and fresh weight of the plant in comparison with the uninoculated treatment (Charoenrak et al., 2019).

Moreover, previous studies affirmed that *Trichoderma* application during the cultivation of lettuce in greenhouses increases the yield, root area and length, photosynthetic pigments such as chlorophyll b and total chlorophyll, photosynthesis rate, water use efficiency, fresh weight and dry weight of aerial parts, and the absorption of different elements such as phosphorus (P), magnesium (Mg), iron (Fe), manganese (Mn), calcium (Ca), copper (Cu), and zinc (Zn) compared with the control (Caruso et al., 2020; Oljira et al., 2020; Saia et al., 2019; Yedidia et al., 2001). In general, these findings indicate that *Trichoderma* spp. application can improve the biochemical and morphological characteristics of horticultural products, and it is known as an influential factor in root system development, nutrient uptake, plant stress response, and the production of secondary metabolites (Szczałba et al., 2019).

Providing nutrients at appropriate levels during crop growth stages increases the yield and quality of lettuce in hydroponic systems (Genuncio et al., 2012; Song, Huang, Hao, et al., 2020; Song, Huang, Song, et al., 2020). Song, Huang, Song, et al. (2020) reported the effect of three levels of Hoagland solution (25%, 50%, and 75%) and photoperiods (12 h/12 h, 15 h/9 h, and 18 h/6 h) in a growth chamber with a hydroponic system on the growth and development of lettuce, showing that the highest number of leaves and fresh and dry weight were observed at 25% concentration under the 18 h/6 h photoperiod. The highest concentrations of P and K were revealed in plants cultivated at the highest concentration of the nutrient solution (50% concentration under 12 h/12 h photoperiod for P and 75% concentration under a 18 h/6 h photoperiod for K, respectively). Based on these results, the lowest nitrate content was observed at 25% concentration under 12 h/12 h photoperiod. Therefore, finding the appropriate level of nutrient solutions plays a vital role in improving the quality and yield of vegetables such as lettuce.

According to extensive studies on the effect of *Trichoderma* on the growth, quality, and yield of different crops in open fields and even greenhouse crops based on solid and mineral substrates (cocopeat and perlite), few studies have been conducted on the role of this fungus in the absorption of nutrients in hydroponic systems (Khan et al., 2021; Li et al., 2015). Therefore, considering the importance of *Trichoderma* spp. as a biostimulant, this study was designed and conducted to investigate the role of *T. harzianum* MVT801 on the growth, yield, nutrient concentration, and physiological characteristics of *L. sativa* “Lollo Rossa” cultivated in a floating system to reduce the use of chemical fertilizers.

Considering the extensive studies on the effect of Trichoderma on the growth, quality, and performance of different crops in open fields and even greenhouse crops based on solid and mineral substrates (cocopeat and perlite), few studies have been conducted on the role of this fungus in nutrient absorption in the floating system.

## 2 MATERIALS AND METHODS

### 2.1 Plant material, cultivation system, and experimental design

**FIGURE 1:**
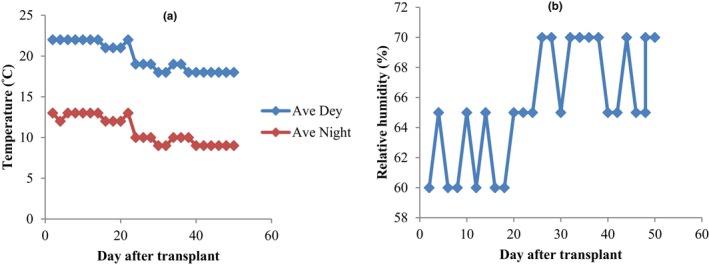
Weather data inside the greenhouse. (a) Average day and night temperature during the growth period. (b) Average relative humidity inside the greenhouse during the growth period.

Seeds of *L. sativa* “Lollo Rosso” (var. Concorde RZ, Rijk Zwaan, Netherland) were germinated at room temperature (20°C) in the laboratory condition (30 ± 5% relative humidity and 240 Lux light intensity with fluorescent lamps). The seeds were germinated in a petri dish with filter paper. Germinated seeds were planted in seedling trays filled with sterile cocopeat (Hexa Growbag, Iran) and perlite (70:30 v:v) and seedlings were produced in greenhouse conditions at the Faculty of Agriculture, Shahid Chamran University of Ahvaz, Ahvaz, Iran (latitude 31°20′ N, longitude 48°41′ E). The seedlings (1 seedling per pot) were transferred (at the 3–4 true‐leaf stage) to the floating system (2 L plastic pots connected to the air pump (output = 280 L/min) with 16 mm drip irrigation pipe, *dropper 2 L per hour, air stone*, and spaghetti tubes and required fitting). Plants were grown under 20/11 ± 2°C (day/night temperature), 65 ± 5% relative humidity, and a natural photoperiod. A factorial experiment based on a completely randomized design with three replications (two plants per replication) was performed from December 2020–February 2021. The weather data inside the greenhouse are shown in Figure 1. Experimental treatments included different ratios of modified Hogland nutrient solution 25% (NR_25_), 50% (NR_50_), and 100% (NR_100_), along with fungal biofertilizer (without *T. harzianum* (T_0_) and with *T. harzianum* (T_1_)). Lettuce plants were grown in the floating system with the following nutrient solutions: N (131), P (62), K (490), Ca (190), Mg (24), Fe (2.236), B (0.324), Mn (0.275), Zn (0.262), Cu (0.048), and Mo (0.048) mg L^−1^ (Tsouvaltzis et al., 2020). Removal of other microorganisms was obtained by passing the purified water (pH = 7.8 and EC = 0.2 dS/m) through antibacterial filters. The pot was disinfected with 10% sodium hypochlorite. Different ratios of nutrient solution, including 25% (NR_25_), 50% (NR_50_), and a complete nutrient solution (100%) (NR_100_), were prepared using purified water, and the pH of the nutrient solutions was also adjusted to 6.0 by nitric acid. For 25% and 50% treatments, 1/4 and 1/2 values of the above elements were used, respectively. Nutrient solutions were poured into pots for each treatment. Fungal biofertilizer (Trichorun P®) in powder form and containing *T. harzianum* MVT801 was supplied by Biorun©, Iran. Fungal treatments were applied 10 days after transplanting the seedlings to the floating system and ensuring the establishment of seedlings. According to the company's recommendation, Trichorun P® (population: 2 × 10^7^ propagol per g of dry matter, EC (1:10) = 2.4 dS m^−1^ and pH (1:10) = 7.2) aqueous suspension was prepared using the nutrient solution of each pot with 8 g of fungal powder. The pots were covered with aluminum foil to prevent the entry of other microorganisms and protect the nutrient solutions.

### 2.2 Plant growth parameters

At the time of harvest (50 days after transplant, DAT), lettuce plants were separated into aerial biomass parts and roots. Fresh weight of shoots (SFW) and roots (RFW), and after oven drying at 70°C for 48 h, related dry weights (SDW and RDW, respectively) were measured by a digital weighing balance. Also, the number of leaves (LN) in each treatment was counted.

### 2.3 Ecophysiological parameters

Photosynthetic traits such as net photosynthesis rate, transpiration rate, and stomatal conductance were measured on complete mature leaves during the growing season twice (35 and 45 days after transplanting) by an LCi‐SD device (UK), and their average was reported. For this purpose, between 10 and 11 am, the developed mature leaves were placed in the device chamber under the natural photoperiod and natural CO_2_ concentration of the greenhouse. Photosynthetic pigments such as chlorophyll a (Cha), chlorophyll b (Chb), total chlorophyll (ChT), and carotenoids (CAR) were measured in fully expanded leaves as reported by Carter and Knapp (Carter & Knapp, 2001) using methanolic extract. Extract absorption was read at 665.2, 652.4, and 470 nm wavelengths by using a spectrophotometer (UV‐1201, Shimadzu, Japan) and calculated using the Lichtenthaler and Buschmann equation (Lichtenthaler & Buschmann, 2001). Water use efficiency (WUE) was obtained from the ratio of dry matter produced to the water consumed (Oljira et al., 2020).

### 2.4 P, K, and nitrate content in plant tissues

Dry leaf samples of lettuce were used for mineral element determination. Plant materials are milled by the electrical grinder. To measure P and K, 1 g of dried samples was digested with 10 mL of nitric acid (65%) for 6 h at 110°C. The completion of the digestion is determined by the signs of the amber‐yellow color of the samples, and the measurements were carried out on the digested samples. Briefly, P concentration was measured by a vanadate‐molybdate method using a spectrophotometer (UV‐1201, Shimadzu, Japan) at a wavelength of 430 nm, and K concentration was measured by a flame photometer device, respectively. Plant nitrate was determined by the rapid colorimetric method using the nitration of salicylic acid (Cataldo et al., 1975). For this purpose, 25 mL of 90°C water was added to 0.1 g of plant sample. After shaking for 30 min, the solution was filtered using filter paper. 0.8 mL of sulfosalicylic acid and 19 mL of 2 N sodium hydroxide were added to 0.2 mL of this extract. Finally, the absorbance of nitrate was examined at 410 nm by a spectrophotometer (UV‐1201, Shimadzu, Japan).

### 2.5 Statistical analysis

The analysis of variance (ANOVA) of the experimental data was evaluated using SAS 9.1 software (SAS Institute, Cary, NC, USA) after checking the data for normality and homoscedasticity through Shapiro–Wilk's test (*p* > .05) and Levene's test (*p* < .05), respectively. Data analysis was performed using the general linear model (GLM) procedure, and means were compared using Duncan's multiple range tests at *p* ≤ .05 for each of the significant variables measured.

## 3 RESULTS

### 3.1 Combined effects of *T. harzianum* and different ratios of nutrient solution

**FIGURE 2:**
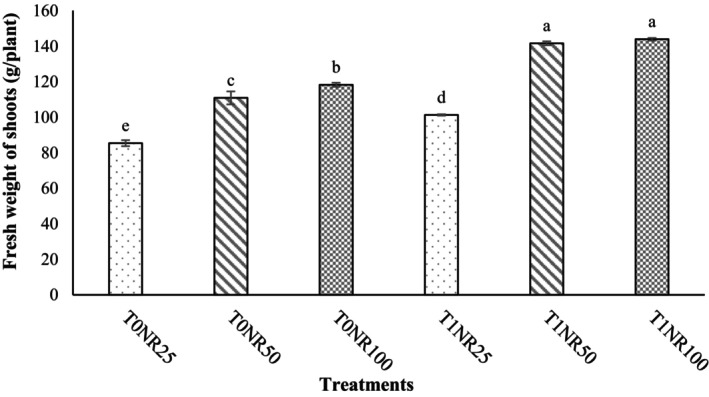
Effect of *Trichoderma harzianum* MVT801 inoculation and different ratios of nutrient solution on the fresh weight of “Lollo Rosso” lettuce shoots (T_0_ and T_1_: uninoculated and inoculated with *Trichoderma*, respectively) and (NR_25_ = 25% nutrient solution, NR_50_ = 50% nutrient solution, NR_100_ = complete nutrient solution). The interactions between the treatments were significant at the level of 1% probability. Means followed by different lowercase letters in a column were significantly different according to Duncan's multiple‐range test (*p* ≤ .05).

**FIGURE 3:**
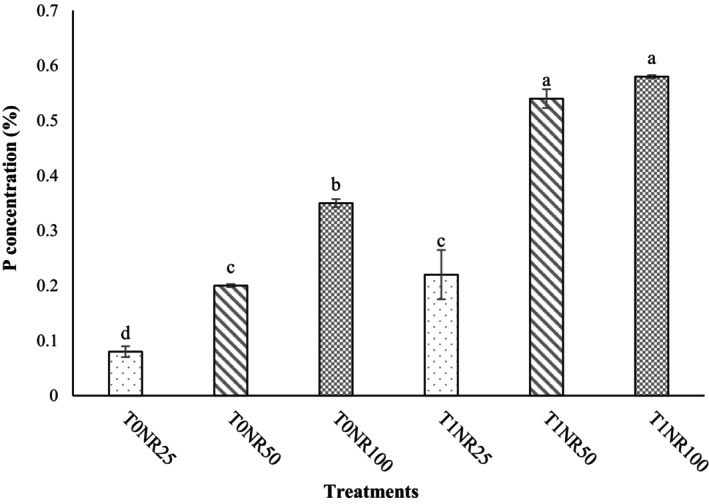
Effect of *Trichoderma harzianum* MVT801 inoculation and different ratios of nutrient solution on phosphorus (P) concentration of “Lollo Rosso” lettuce leaves (T_0_ and T_1_: uninoculated and inoculated with *Trichoderma*, respectively) and (NR_25_ = 25% nutrient solution, NR_50_ = 50% nutrient solution, NR_100_ = complete nutrient solution). The interactions between the treatments were significant at the level of 1% probability. Means followed by different lowercase letters in a column were significantly different according to Duncan's multiple‐range test (*p* ≤ .05).

**FIGURE 4:**
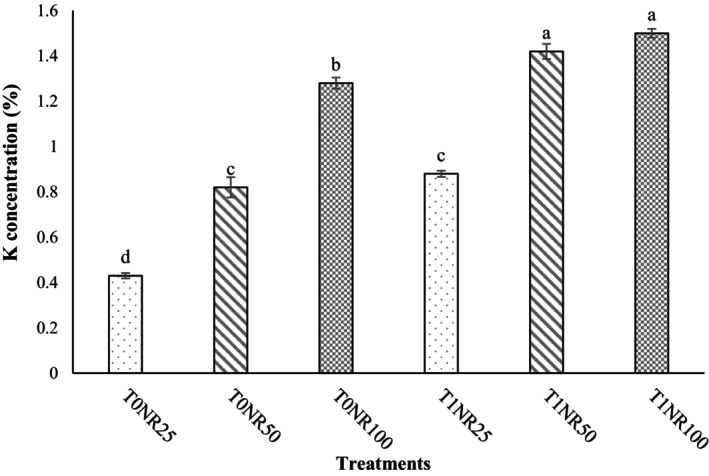
Effect of *Trichoderma harzianum* MVT801 inoculation and different ratios of nutrient solution on potassium (K) concentration of “Lollo Rosso” lettuce leaves (T_0_ and T_1_: uninoculated and inoculated with *Trichoderma*, respectively) and (NR_25_ = 25% nutrient solution, NR_50_ = 50% nutrient solution, NR_100_ = complete nutrient solution). The interactions between the treatments were significant at the level of 1% probability. Means followed by different lowercase letters in a column were significantly different according to Duncan's multiple‐range test (*p* ≤ .05).

The interaction between *T. harzianum* and nutrient solution (*T. harzianum* × Nutrient solution) was significant on dry weight of shoots, dry weight of roots, number of leaves, photosynthesis rate, stomatal conductance, transpiration rate, nitrate content, water use efficiency (Table 1), fresh weight of shoots (Figure 2), and uptake of minerals such as P (Figure 3) and K (Figure 4). The results of this experiment also clearly showed that in plants inoculated with *T. harzianum*, despite a 50% reduction in nutrient solution ratio, there was no significant difference in leaf number, yield, photosynthetic indices, and nutrient uptake of “Lollo Rosso” leaves compared with a complete nutrient solution treatment (NR_100_), which is one of the notable results of this study.

**TABLE 1:** The interaction effect of *Trichoderma harzianum* MVT801 and different ratios of nutrient solution (NR: 25%, 50%, and 100%) on dry weight of shoots (SDW), dry weight of roots (RDW), number of leaves (LN), photosynthesis rate, stomatal conductance, transpiration rate, nitrate content, and water use efficiency (WUE) in *L. sativa* “Lollo Rosso” plants under floating cultivation.

Treatment	SDW (g)	RDW (g)	LN	Photosynthesis rate (μmol/m^2^s)	Stomatal conductance (mmol/m^2^s)	Trenspiration rate (mmol/m^2^s)	Nitrate (ppm)	WUE (g/L)
T_0_NR_25_	9.46^e^	1.38^d^	18.00^d^	0.53^d^	0.05^c^	1.03^d^	336.55^b^	2.36^e^
T_0_NR_50_	10.76^c^	2.02^c^	22.00^b^	1.68^c^	0.07^c^	1.44^c^	343.75^a^	2.69^c^
T_0_NR_100_	10.97^b^	2.10^b^	23.00^b^	2.37^b^	0.12^b^	1.98^b^	343.88^a^	2.74^b^
T_1_NR_25_	10.53^d^	1.98^c^	19.33^c^	1.60^c^	0.08^c^	1.46^c^	342.35^a^	2.63^d^
T_1_NR_50_	11.60^a^	3.08^a^	26.33^a^	2.90^a^	0.16^a^	2.51^a^	341.21^a^	2.90^a^
T_1_NR_100_	11.63^a^	3.10^a^	26.66^a^	2.94^a^	0.17^a^	2.59^a^	342.08^a^	2.91^a^
Significance	**	**	**	*	*	**	*	**

*Note*: The statistical relevance is provided (* = *p* ≤ .05; ** = *p* ≤ .01). Means followed by different lowercase letters in a column were significantly different according to Duncan's multiple‐range test (*p* ≤ .05). (T_0_ and T_1_: uninoculated and inoculated with *Trichoderma*, respectively) and (NR_25_ = 25% nutrient solution, NR_50_ = 50% nutrient solution, NR_100_ = complete nutrient solution).

**FIGURE 5:**
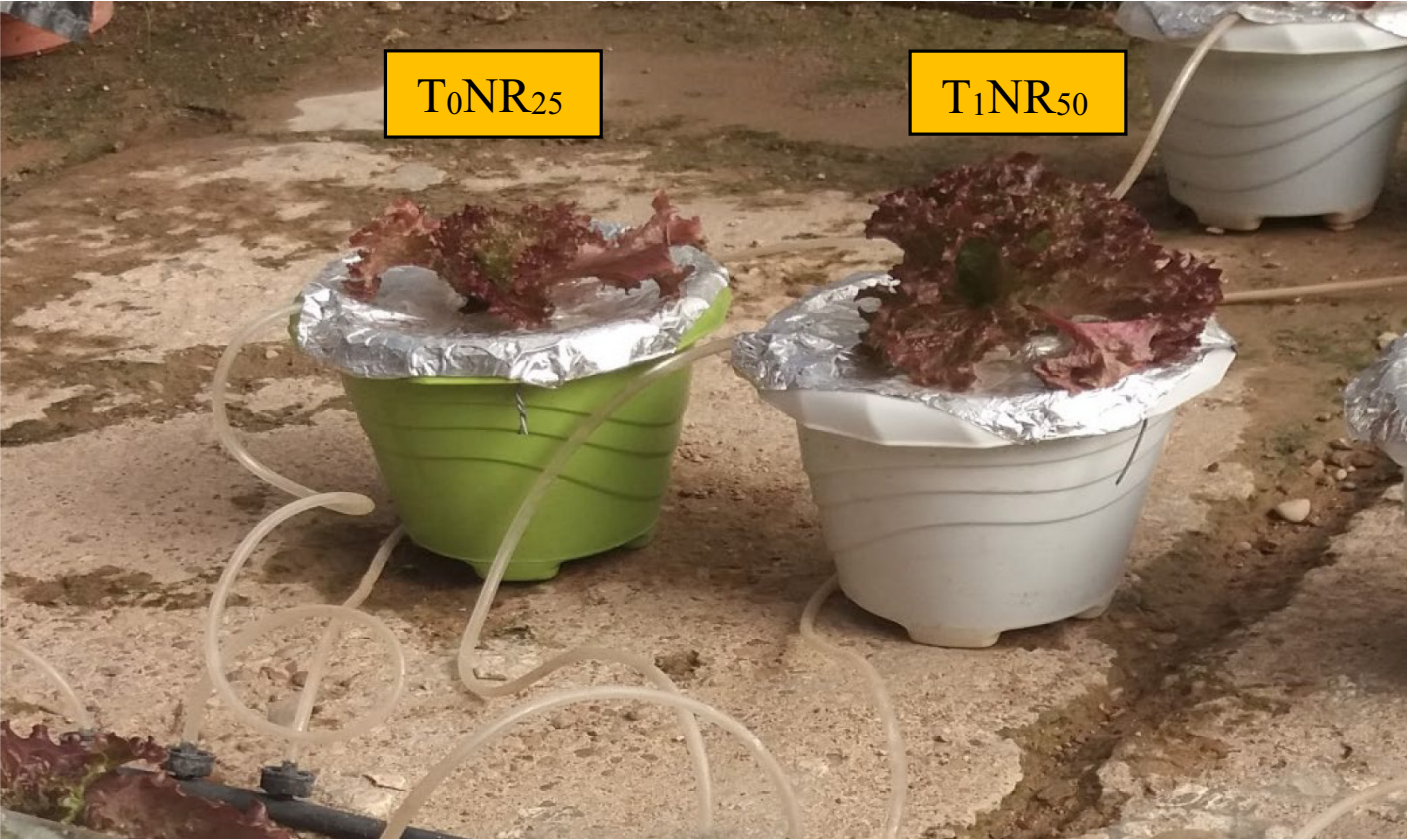
Effect of *Trichoderma harzianum* MVT801 inoculation and different ratios of nutrient solution on the fresh weight of “Lollo Rosso” lettuce shoots (T_0_ and T_1_: in‐inoculated and inoculated with *Trichoderma*, respectively) and (NR_25_ = 25% nutrient solution, NR_50_ = 50% nutrient solution). The T_1_NR_50_ treatment, compared with the T_0_NR_25_ treatment, increased the yield by 65.80%.

In this regard, the highest amount of fresh weight of the aerial part was observed in the T_1_NR_50_ and T_1_NR_100_ treatments, which were 18.62% and 13.77% higher than the treatments without *Trichoderma* (T_0_NR_50_ and T_0_NR_100_), respectively (Figure 2). Also, the T_1_NR_50_ treatment compared with the T_0_NR_25_, T_0_NR_50_, and T_0_NR_100_ treatments increased the yield by 65.80%, 18.62%, and 12.33%, respectively (Figure 5). Also, the highest concentrations of P and K in “Lollo Rosso” leaves were observed in T_1_NR_50_ and T_1_NR_100_ treatments, which, in comparison with T_0_NR_50_ and T_0_NR_100_ treatments, caused an increase of 170% and 65.71% of P concentrations and 73.17% and 17.18% of K concentrations, respectively (Figures 3 and 4). As it is known, the effect of inoculating *T. harzianum* in 50% of the nutrient solution was more tangible than in the complete solution (100%). The lowest nitrate concentration was observed in the treatment without *Trichoderma* inoculation and a 25% nutrient solution. Also, nitrate concentration in inoculated plants was lower than uninoculated plants, although this difference was insignificant. WUE in plants inoculated with *Trichoderma* was higher than in plants without inoculation, and no significant difference was observed between inoculated treatments and the 50% ratio and complete nutrient solution (Table 1). Also, inoculation of *Trichoderma* in low ratios of nutrient solution (T_1_NR_25_) could not increase the WUE compared with inoculated treatments and ratios of 50% and 100% of nutrient solution (T_0_NR_50_ and T_0_NR_100_) (Table 1).

### 3.2 Effects of *T. harzianum*

Plants inoculated with *T. harzianum* showed an increase equal to 24.87% in fresh weight of roots (RFW) compared with uninoculated plants (Table 2). Also, the findings of this study indicated that the use of biofertilizer containing *T. harzianum* MVT801 only increased photosynthetic pigments such as Chb and ChT and had no effect on Cha and CAR (Table 2).

**TABLE 2:** The main effects of *Trichoderma harzianum* MVT801 and different ratios of nutrient solution (NR: 25%, 50%, and 100%) on fresh weight of roots (RFW), chlorophyll a (Cha) and chlorophyll b (Chb), chlorophyll total (ChT), and carotenoids (CAR) of *L. sativa* “Lollo Rosso” under floating cultivation.

Treatment	RFW (g)	Cha (mg/g)	Chb (mg/g)	ChT (mg/g)	CAR (mg/g)
T					
T_0_	26.33	0.48	0.32	0.81	3.93
T_1_	32.88	0.71	0.51	1.22	4.39
Significance	**	ns	**	*	ns
NR					
NR_25_	23.68^c^	0.50	0.35	0.86	3.82
NR_50_	31.96^b^	0.58	0.41	1.00	4.19
NR_100_	33.16^a^	0.70	0.48	1.18	4.47
Significance	**	ns	ns	ns	ns

*Note*: *p ≤ .05 and **p ≤ .01. Means followed by different lowercase letters in a column were significantly different according to Duncan's multiple‐range test (*p* ≤ .05). T_0_ and T_1_ = uninoculated and inoculated with *Trichoderma*, respectively, and NR_25_ = 25% nutrient solution; NR_50_ = 50% nutrient solution; NR_100_ = complete nutrient solution.

Abbreviation: ns, not significant.

### 3.3 Effects of different ratios of nutrient solution

Findings of this experiment indicated that the use of different ratios of nutrient solution had a significant effect on the fresh weight of roots (*p* < .01). NR_50_ and NR_100_ increased the fresh weight of roots by 34.96% and 40.03%, respectively, compared with NR_25_ (Table 2). Using nutrient solutions in different ratios had no significant effect on photosynthetic pigments (Table 2).

## 4 DISCUSSION

Our experiment exhibited a positive effect of *T. harzianum* MVT801 inoculation on growth and yield, pigments (chlorophyll b and total chlorophyll), photosynthetic indices, and P and K concentrations of “Lollo Rosso” lettuce leaves. Similar results have been reported by Martins Filho et al. (2019) and Rouphael et al. (2020). These authors showed that *T. harzianum* increased lettuce leaf area, root length, and shoot and root dry matter compared with the control. Increased yield with *T. harzianum* inoculation has been observed in many other horticultural crops, such as cucumbers, eggplants, peas, peppers, radishes, tomatoes, and carrots (Martins Filho et al., 2019). According to these findings, our positive results are probably due to the ability of *T. harzianum* to improve nutrient uptake, induce disease resistance, and enhance plant growth (Srichamnong et al., 2021). On the other hand, the better growth recorded in plants inoculated with *Trichoderma* may be partly related to their better water status and the influential role of fungal hyphae in increasing nutrient solution uptake. Recent studies highlighted that microorganisms such as fungi and bacteria are capable of producing hormones, vitamins, enzymes, and other compounds that enhance plant growth, which can increase the availability and absorption of nutrients and affect plant growth and yield (López‐Bucio et al., 2015; Stojanović et al., 2020). Increased root growth and leaf number have been shown by applying *T. harzianum* at in vitro conditions (Sofo et al., 2010). As the previous findings showed, *Trichoderma*, by producing hormone‐like compounds such as indole‐3‐ethanol (IEt), indole‐3‐acetic acid (IAA), and indole‐3‐acetaldehyde (IAAld), stimulates the formation of lateral roots, which improves root surface area and establishes favorable conditions for fungal colonization, as well as indirectly increases water and nutrient absorption and processes related to photosynthesis (Carillo et al., 2020). The efficiency of *Trichoderma* spp. depends on plant growth, plant species, ability to colonize roots, and type of culture medium (Stojanović et al., 2020). Several reports have shown that some species of *Trichoderma* spp., including *T. atroviride*, *T. koningii*, *T. harzianum*, and *T. virens*, are among the biostimulants that improve crop yield, nutrient efficiency, and tolerance to abiotic stresses (Colla et al., 2015; Fiorentino et al., 2018; Formisano et al., 2021; Saia et al., 2019). Direct and indirect mechanisms of the effect of biostimulation of *Trichoderma* strains include (1) improving lateral root growth, (2) induction of active plant proteins, and (3) production and rhizosphere secretion of auxins and secondary metabolites such as volatile and non‐volatile substances that stimulate various reactions of plants and increase nutrient uptake and crop productivity (Bonini et al., 2020). Various reasons have been reported by researchers for increasing P uptake in the presence of *Trichoderma*, which will be discussed further.

In hydroponic systems, nutrient solutions improve and increase the yield and quality of vegetables such as basil, lettuce, and spinach. In agreement with our data, Song, Huang, Hao, et al. (2020) reported that lettuce biometric traits and photosynthetic indices were more affected by different ratios of nutrient solution than photosynthetic pigments. Also, Geng et al. (2022) showed that *T. harzianum* reduced stomatal closure and increased the net photosynthesis rate and the activity of RuBisCO and FBPase in tomato plants. These positive characteristics can be affected by the importance of P in the photosynthetic chain of plants, as in the present study, inoculated plants showed a significant amount of P, fresh and dry weight of shoots, and number of leaves compared with uninoculated plants (Figures 2 and 3 and Table 1).

The interaction of different nutrient solution ratios and *T. harzianum* showed that inoculated treatments had higher growth, photosynthetic, and nutrient uptake indices than non‐inoculated treatments. The findings of this experiment showed that *T. harzianum*, by affecting the absorption and transport of elements in plants with half the concentration of nutrient solution, had a significant contribution to improving growth and physiological characteristics compared to the treatment with 100% nutrient solution. The fungus possesses incredible potential for not only enhancing the plant's physiological uptake mechanisms but also playing a direct role in the solubilization of various nutrients in the rhizosphere. These nutrients, such as rock phosphate, iron, copper, and divalent ions, are vital for plant growth and development. Furthermore, the fungus has the ability to produce siderophores that facilitate the availability of these elements to the plant (Harman et al., 2004). Regarding the yield, results indicated that no significant difference was observed between the 50% and 100% nutrient solution treatments in lettuce inoculated with *T. harzianum*. The findings of Rouphael et al. (2020) showed that *T. harzianum* stimulates root growth in the rhizosphere and changes in root structure, which include morphological changes that affect the absorption of nutrients, especially nitrate, calcium, magnesium, and potassium, and ultimately affect overall yield. Ion concentration and nutrient flow rate determine nutrient availability and plant water uptake in hydroponic systems. The ion concentration of the nutrient solution is a determining factor in the regulation of the stoma ostiole (through osmotic potential), photosynthetic efficiency, leaf expansion, leaf and root growth, as well as in the crop harvest index in hydroponic cultivation. The uptake of water and nutrients by plants is related to the ion concentration of the nutrient solution, and the results are related to the accumulation of fresh weight or fresh plant mass of hydroponic lettuce due to the appropriate osmotic potential of the nutrient solution (Genuncio et al., 2012). Therefore, it seems that *T. harzianum*, by affecting the absorption properties of elements in the roots of plants with lower concentrations (50%), has created similar conditions with the treatment of 100% nutrient solution.

According to the results, the dry weight of roots and shoots was affected by different concentrations of nutrients and inoculation of *T. harzianum*. The improvement of biometric indices of “Lollo Rosso” lettuce after inoculation with *T. harzianum* can be affected by the increase in root absorption efficiency and the increase in root dry weight, which is consistent with the latest report (Formisano et al., 2021). The improvement of biometric parameters can also be due to signal molecules secreted by fungal mycelium, such as hormonal compounds (i.e., ethylene and auxin), low‐molecular‐weight peptides, and volatile organic compounds (Salwan et al., 2019), which re‐modulate gene expression and biochemical processes (Carillo et al., 2020). These compounds not only increase the uptake of micronutrients (i.e., Fe, Mn, and Zn) and macronutrients (i.e., P, Mg, Ca, and K) in the roots but also stimulate root growth and plant development (Salwan et al., 2019; Vinale et al., 2013). The latest scientific studies showed that *T. harzianum* improves the growth and nutrition of lettuce and monocot seedlings in hydroponic systems at intervals of 50 and 28 days, respectively (Moreira et al., 2022; Yasmeen & Siddiqui, 2018).

Increases in P and K concentrations in tomato seedling shoots inoculated with two *T. harzianum* strains (T447 and T969) have been previously reported (Azarmi et al., 2011). Further assimilation and transfer of P and Mg in plants inoculated with *Trichoderma* increases the net CO_2_ assimilation rate and quantum efficiency of open photosystem II and subsequently improves vegetative growth (Bonini et al., 2020), which is consistent with the results of this study. Previous reports have also shown that photosystem II is protected from photoinhibition by regulating gene expression in photosynthetic processes, in which Mg and P play a vital role (Hernández & Munné‐Bosch, 2015). Plants inoculated with *Trichoderma* improve photosynthetic processes through upregulation of gene and chloroplast components (Harman et al., 2021). Therefore, in plants inoculated with *Trichoderma*, the role of element uptake like P and K in increasing photosynthesis, growth, and yield is undeniable. Various reasons have been reported by researchers for increasing P uptake in the presence of *Trichoderma*. The production of the phosphatase enzyme by *Trichoderma asperellum* Q1 to increase the solubility of P under salinity stress in *Arabidopsis* has been reported (Lei et al., 2017). Tandon et al. (2020) stated that in the presence of *Trichorderma*, lowering of pH and organic acid production is the primary mechanism of P solubilization under abiotic stress conditions. Li et al. (2015) showed that *Trichoderma* mediated P solubilization through hydrolysis (ferric reductase and phytase activity), chelation (siderophore production), and redox.

The presence of a significant difference in the nitrate concentration of the leaves of the 25% treatment compared with the 50% and 100% treatments can be due to the low concentration of nitrate ions in the nutrient solution. Also, the findings of this study indicated that there is no significant difference in leaf nitrate content between the 50% and 100% nutrient solutions of inoculated and uninoculated plants. However, a slight decrease in nitrate concentration was observed in both the 50% and 100% treatments colonized with *Trichoderma* compared with the uninoculated treatment. It seems that a slight decrease in the nitrate content of T_1_NR_50_ treatment (341.21 ppm) compared with T_0_NR_50_ treatment (343.75 ppm) was able to improve the growth of inoculated plants through the conversion of nitrate to nitrogen‐containing structures such as amino acids and secondary metabolites. Previous findings showed that *Trichoderma* can improve nitrogen metabolism by increasing asparagine content, which is the most common amide used for nitrogen transport in plants. This ability is induced by *Trichoderma* either by increasing the expression of enzymes such as nitrate reductase or by promoting nitrogen uptake (Carillo et al., 2020; Harman et al., 2004).

## 5 CONCLUSIONS

Our experiment on “Lollo Rosso” lettuce confirmed the ability of *T. harzianum* MVT801 to increase crop productivity, photosynthetic efficiency, and nutrient uptake compared with the uninoculated plants in the floating system. Concomitant use of *T. harzianum* with different ratios of nutrient solution showed that despite a 50% reduction in nutrient solution, no significant differences were observed with full‐strength nutrient solution in yield, shoot and root dry weight, leaf number, photosynthetic indices, and leaf phosphorus and potassium concentrations, which is a remarkable result of this study. Therefore, in order to reduce the use of chemical fertilizers in soilless cultures, such as the floating system, the use of biostimulants like *T. harzianum* can be an effective option to help preserve the environment in addition to increasing crop productivity. However, it seems that to achieve comprehensive results, more studies at the cellular and molecular level are needed so that it can be more decisively promoted among farmers and floating producers.

## AUTHOR CONTRIBUTIONS

**Edris Shabani:** Conceptualization (lead); data curation (equal); funding acquisition (lead); investigation (lead); methodology (lead); project administration (lead); resources (equal); software (equal); supervision (lead); validation (equal); visualization (equal); writing – original draft (lead); writing – review and editing (lead). **Naser Alemzadeh Ansari:** Methodology (equal); resources (equal). **Mohammad Reza Fayezizadeh:** Data curation (equal); formal analysis (equal); software (equal); validation (equal); visualization (equal). **matteo caser:** Writing – review and editing (supporting).

## FUNDING INFORMATION

This research received no external funding.

## CONFLICT OF INTEREST STATEMENT

The authors declare no conflict of interest.

## Data Availability

The data are confidential.
